# Pragmatic randomised controlled trial to evaluate the clinical and cost-effectiveness of compression therapy for surgical wounds healing by secondary intention following excision of lower limb keratinocyte cancers in UK skin cancer surgery centres (HEALS2 trial): study protocol

**DOI:** 10.1136/bmjopen-2025-115191

**Published:** 2026-09-07

**Authors:** Henrietta Konwea, Myka Ransom, Rachel Abbott, Iqra Ashraf, Chris Bojke, Sarah Tess Brown, Howard Collier, Joanna Connor, Rachael Mary Gilberts, Jasmine Mann, Annah Muli, Jane Nixon, Emma Pynn, David Veitch, A G H Wernham, Elizabeth McGinnis

**Affiliations:** 1 Academic Unit of Health Economics, Leeds Institute of Health Sciences, University of Leeds, Leeds, UK; 2 Leeds Institute of Clinical Trials Research, University of Leeds, Leeds, UK; 3 University Hospital of Wales Healthcare NHS Trust, Cardiff, Wales, UK; 4 South Warwickshire University NHS Foundation Trust, Warwick, UK; 5 HEOR, Lumanity, Sheffield, UK; 6 Homerton Healthcare NHS Foundation Trust, London, UK; 7 Aneurin Bevan University Health Board, Abergavenny, Wales, UK; 8 Department of Dermatology, St Michael’s Clinic, Shrewsbury, UK; 9 Department of Dermatology, Royal Wolverhampton NHS Trust, Wolverhampton, UK; 10 Department of Dermatology, Walsall Healthcare NHS Trust, Walsall, UK

**Keywords:** Dermatological tumours, Surgical dermatology, WOUND MANAGEMENT

## Abstract

**Introduction:**

Keratinocyte cancers (KCs) are increasing in incidence in the UK, with over 234 000 cases annually, largely due to demographic changes and sun exposure. Skin cancers, including malignant melanoma and KCs, are projected to cost the NHS over £180 million annually and impose healthcare demands. Although KCs have high cure rates following surgical excision and primary closure, wounds healing by secondary intention (HBSI) particularly on the lower leg present clinical challenges. Compression therapy (CT) known to improve venous insufficiency and venous ulcer healing may also benefit HBSI following lower limb KC excision but current evidence is sparse.

**Methods and analysis:**

**H**ealing of **E**xcision**A**l wounds on **L**ower legs by **S**econdary intention (HEALS2) is a multicentre, prospective, Phase III, parallel group, open-label, randomised controlled trial with embedded internal pilot and blinded endpoint assessment. A total of 396 participants (≥18 years) from UK skin cancer surgery centres will be randomised 1:1 to assess the clinical and cost-effectiveness of standard care (SC) alone or with CT (SC+CT) for surgical wounds HBSI following excision of lower limb KCs.

The primary outcome is time to healing from randomisation (maximum 52-week follow-up). Secondary outcomes include incidence of infection, antibiotic usage, scar quality, safety (including complications and hospitalisations), quality of life, cost-effectiveness and relationship between post-partial closure wound area, type of partial closure method and time to healing. Exploratory objectives include association between short-term wound area reduction and time to HBSI, patient acceptability, adherence and associations between wound breakdown post-healing and CT use. A qualitative sub-study will explore patients’ experiences and decision-making during CT use.

Primary endpoint analysis will be conducted on the intention-to-treat population using a multivariable Cox Proportional Hazards modelling. Secondary outcomes and safety will be evaluated using other regression models and descriptive statistics, respectively. Cost-effectiveness will be evaluated using within-trial analysis and decision-analytic modelling. Patient and public involvement representatives are contributing to trial design and oversight. The study protocol complies with the Standard Protocol Items: Recommendations for Interventional Trials (SPIRIT) Statement.

**Ethics and dissemination:**

Ethics approval has been granted by the Yorkshire & The Humber – Leeds East Research Ethics Committee (ref 23/YH/0247). Findings will be published in high-impact peer-reviewed journals.

**Trial registration number:**

ISRCTN12417689.

**STRENGTHS AND LIMITATIONS OF THIS STUDY**

- A multicentre randomised controlled trial investigating the effects of adjunctive compression therapy (CT) in patients following surgical excision of keratinocyte cancer (KC) and healing by secondary intention.
- Study includes patients’ perspective of the experience of CT post-surgical excision of KC.
- Pragmatic trial with choices of evidence-based CT that consider patient abilities and lifestyles and accommodate different local clinical pathways.
- Limited to patients with secondary intention healing (excludes those who have primary closure, flap or graft).
- Limited to those who have a suspected KC (non-KCs for example, melanoma usually experience a different clinical pathway).

## Introduction

 Skin cancers are common with numbers increasing yearly due to demographic changes, for example, an increasingly aged population and social changes including levels of sun exposure.^1^ Skin cancers, including malignant melanoma and keratinocyte cancers (KCs), in the UK are projected to cost the NHS over £180 million per year and impose significant demands on primary, community and secondary care services.^2 3^ KCs, including basal cell and squamous cell carcinomas, are very common (annual incidence of over 234 000 cases)^4^ but have high cure rates with surgical excision and primary closure/skin graft/flap or partial/no closure and healing by secondary intention (HBSI).

Surgical wounds healing after excision of KC lesions on the lower leg provide particular clinical challenges. First, due to anatomical location and lack of skin laxity, many are not amenable to primary closure.^5^ Second, people undergoing surgery for KC on the lower leg are typically elderly with concurrent mobility limitations, peripheral vascular disease and local oedema, factors which delay healing and increase risk of complications including infection.^6 7^ Thirdly, postoperative oedema due to the inflammatory process is a common sequela of surgical wounds which normally resolves within a few days in most body sites. However, in the lower leg, the cumulative effect of peripheral vascular/venous disease and gravity delays this resolution and is likely to compromise wound healing.

The annual prevalence of acute and chronic wounds increased by 71% between 2012/13 and 2017/2018, with an estimated 3.8 million patients with a wound (excluding surgical wounds healing within 4 weeks) managed by the National Health Service (NHS) in 2017/18 and an annual cost of £8.3 billion.^8^ Surgical wounds (of over 4 weeks) accounted for 14% of these wounds, and in turn those on the lower limb account for 15% of all surgical wounds HBSI.^7^ Wounds HBSI impact on quality of life, with considerable patient burden due to pain, exudate, repeated visits for wound dressings, possible infections and inability to work and/or socialise with long term effects of scarring and discomfort.^9^

In considering this patient cohort and common condition, three factors impeding understanding of best practice were identified: a lack of outcome data as patients are usually discharged from secondary care follow-up before healing, an absence of a recognised guideline for the postoperative management and clinical uncertainty about the use of compression as a primary postoperative intervention to promote time to healing and reduce complications.

The lack of evidence to underpin practice was acknowledged by the UK Dermatology Clinical Trials Network (UKDCTN) who supported a programme of research to inform this grant application. The work has included: a 2014^5^ and 2021 survey of clinicians;^10^ UKDCTN-funded trainee-led cohort feasibility study;^11 12^ UKDCTN patient and public involvement (PPI) focus group and a trainee-led systematic review^6^ as detailed in the following sections.

### Patient burden

The UKDCTN-funded Healing of ExcisionAl wounds on Lower legs by Secondary intention (HEALS) cohort study (n=53) was undertaken to assess the feasibility of performing a large-scale definitive phase III trial and is the first study to investigate time to healing, infection and serious adverse events (SAEs) following excision of KC on the lower leg and HBSI in patients without compression.^11 12^ The cohort study recruited from nine centres across the UK. Participants were adults, without significant arterial disease (ABPI ≥0.8), who had planned excision of KC on the lower leg and expected to have a wound HBSI without postoperative compression.

Participants and non-registered patients shared similar demographic characteristics, demonstrating that the sample was representative of the target patient population. Patients received routine follow-up care and data was collected weekly either at standard care (SC) clinic visits or phone calls. Variables collected postoperatively included details of clinical assessments, wound treatments, medications and healing status. Patient pathways were characterised by the frequency of clinical visits, type of professional and care setting for follow-up contacts.

The study population had a median age of 81 years. 21% had mobility limitations and while patients with severe arterial disease (ABPI <0.8) were excluded, 73.6% had visible or palpable signs of venous disease. Reflecting risk factors for delayed wound healing, the study identified considerable patient and NHS burden with median time to healing 81 days (95% CI 73 to 92), incidence of wound infection 30.2% (n=16), hospitalisation for infection/cellulitis 5.7% (n=3), unhealed at 6 months 7.5% (n=4) with healing status unknown at 6 months post-surgery for 7.5% (n=4).^11^

### Evidence for compression therapy and knowledge gap

Compression therapy (CT) has been established by primary research/systematic review evidence as the primary/first-line treatment strategy for healing of venous leg ulcers (VLUs). The VenUS trials have also helped inform which compression systems are most effective.^13 14^ Compression is now commonly used for the treatment of VLUs where it has transformed management, thereby reducing the burden of wounds.

Mechanistically, compression reduces oedema and improves venous return and tissue oxygenation. Given the high levels of underlying venous disease and the normal occurrence of postoperative oedema due to the inflammatory process it is proposed that lower leg surgical wounds HBSI may benefit from compression in a similar manner. However, it is also recognised that CT can be resource intensive (although the use of compression hosiery is now enabling patient self-care) and can sometimes be difficult to apply and uncomfortable for the patient. When our patient group were presented with different compression options, most members agreed that bandages might be hot and/or uncomfortable, but those who had experienced oedema/infection or delayed healing were less concerned with these issues.^12 13^

We conducted a systematic literature review^7^ and did not find any randomised controlled trials (RCTs) comparing no compression with compression for wounds HBSI after excision of KC on lower leg. We identified only two cohort studies with reported median time to healing for no compression (n=53) of 81 days^12^ and mean time to healing with compression (n=10) of 50 days.^15^ Unpublished local audit data at a participating centre also reported 88% (22/25) of patients with compression healed within 6 weeks. From a safety perspective, we also identified a retrospective cohort n=366^16^ which reported that patients who had postoperative compression were less likely to develop complications versus those without compression (OR 0.67: p=0.74), but time to healing was not reported.

CT has been rigorously evaluated in the VLU population^17^ with significant evidence that it increases healing rates when compared with no compression, with improved time to healing (five studies; 733 participants; HR 2.17 (95% CI 1.52 to 3.10)) and complete healing (eight studies; 1120 participants: relative risk/risk ratio (RR) 1.77 (CI 1.41 to 2.21)) with no impact on adverse events (AEs).^14^ Systematic review evidence and trials also indicate that: multi-component systems are more effective than single component systems; elasticated systems are more effective than non-elastic, and within Class bandage systems and hosiery are similarly effective, for example, 4-layer bandage, 2-layer bandage and 2-layer hosiery^18^ which has improved patient and clinician choice and associated adherence. For example, the most recent Health Technology Assessment study^13^ found no difference in healing between (Class 3) 4-layer high compression bandaging or 2-layer hosiery, while hosiery was easier to apply, less bulky and use more likely to be sustained.

Based on the perspective of the VLU patient population, there is evidence that some patients do not always adhere to their CT. A qualitative study has been recently published which explored barriers and facilitators to CT.^19^ Findings suggest that adherence is multi-factorial with issues such as the choice of therapy, patient lifestyle and service organisation being contributing factors.

CT is sometimes applied to reduce leg oedema and promote healing for lower limb KC wounds HBSI, but there is clinical equipoise, variation in practice when used and no supporting evidence of effectiveness. Our PPI work has also shown that compression is acceptable to this group of patients. Given the findings from the literature, the feasibility study and with the similarities with mechanistic mode of action in venous conditions, it is therefore essential to assess the clinical and cost-effectiveness of compression as a primary postoperative intervention in terms of reduced time to healing and secondary outcomes including infection.

## Objectives

The aim of this pragmatic RCT is to evaluate the clinical and cost-effectiveness of SC+CT in the healing of surgical wounds HBSI following excision of lower limb KCs.

### Primary objective

To compare the time to HBSI from randomisation, between SC and SC+CT.

#### Secondary objectives

To compare groups for:

- Incidence of infection as measured by modified Bluebelle Wound Healing Questionnaire (WHQ)^20^ until healing.
- Numbers of days participants prescribed antibiotics until healing.
- Scar quality as measured by Patient and Observer Scar Assessment Scale (POSAS).^21^
- Safety events including related complications and hospitalisations until healing (maximum 52 weeks post randomisation).
- Cost-effectiveness via a within-trial analysis and decision analytic model (DAM) assessed from a payer perspective measuring patient health-related quality of life in terms of Quality Adjusted Life Years (QALYs) meeting updated Consolidated Health Economic Evaluation Reporting Standards (CHEERS) 2022 updated reporting guidance.^22^
- Resource use and health-related quality of life (HRQoL) as measured by EuroQol 5 Dimensions 5 Levels questionnaire (EQ-5D-5L) to 26 weeks post randomisation (52 weeks where applicable, for unhealed participants only).
- The relationship between post-partial closure wound area, type of partial closure method, and time to healing.

### Exploratory objectives

These include exploring:

- Associations between short-term wound area reduction between baseline and 4 weeks post randomisation and time to HBSI.
- Patient acceptability and factors affecting adherence to randomised treatment.
- Associations between wound breakdown post healing and the use of CT.

### Qualitative sub-study objectives

These include understanding and assessing:

- Challenges and difficulties encountered during CT.
- Impact of CT on daily activities and quality of life.
- Patients’ satisfaction with the support provided.
- Factors influencing patients’ decisions to continue or discontinue CT.

## Methods

**Figure 1:**
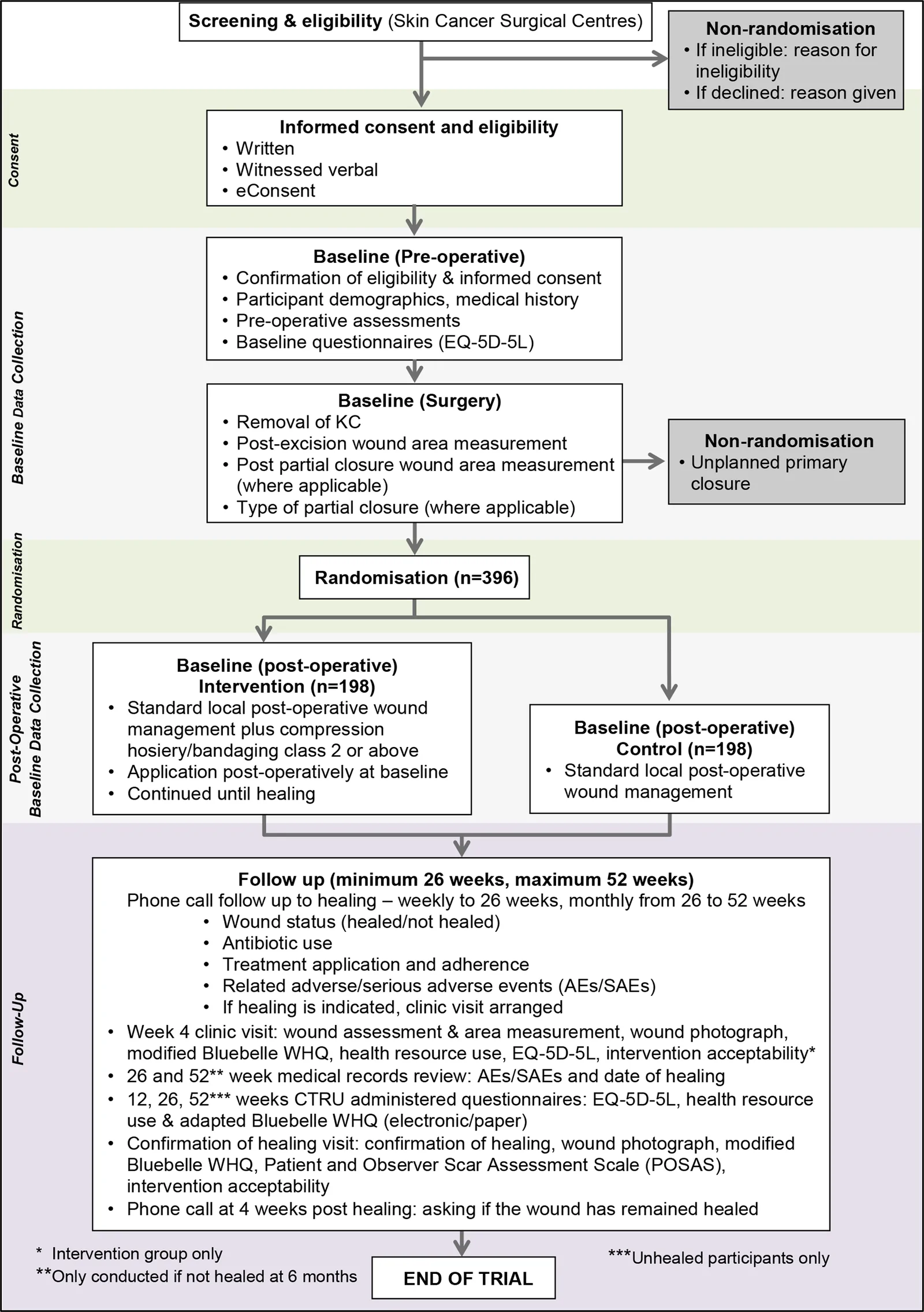
Flow diagram of HEALS2 study design. AE, adverse event; CTRU, Clinical Trials Research Unit; EQ-5D-5L, 5-Level version of EuroQol 5-Dimensions Questionnaire; KC, keratinocyte cancer; POSAS, Patient and Observer Scar Assessment Scale; SAE, serious adverse event; WHQ, Wound Healing Questionnaire.

HEALS2 is a multicentre, prospective, Phase III, parallel group, open-label, RCT with embedded internal pilot, blinded endpoint assessment, economic evaluation, minimum 26-week/maximum 52-week follow-up, comparing time from randomisation to complete surgical wound healing (epithelialisation) by secondary intention between control (SC) and intervention (SC+CT) groups (see study design illustrated in figure 1). The qualitative sub-study entails virtual semi-structured interviews with participants randomised to CT. These will be audio-recorded, transcribed and analysed.

### Eligibility

All adult patients attending a UK secondary care skin cancer surgery centre (SCSC) will be screened for suitability according to the eligibility criteria (box 1), by the attending clinical/research team if they have a suspected KC on the lower leg which is planned to be excised and HBSI. The lower leg is defined as ‘below the knee, including the ankle and foot but excluding the toes’. Patients will be consented preoperatively. Screening logs will be maintained to review reasons not eligible or unable/declined consent and to demonstrate the representativeness of the study sample.

Box 1 **Eligibility criteria**

**Inclusion criteria**

- Aged ≥18 years.
- Planned excision of suspected keratinocyte cancer (KC) on lower leg with healing by secondary intention.
- Ankle-brachial pressure index ≥0.8 or toe pressure of >60 mm Hg (measurements taken within 3 months of randomisation).
- Informed written/witnessed verbal/eConsent.

**Exclusion criteria**

- Planned primary closure/skin graft/flap.
- Receiving/planned compression for another indication.
- Severe venous incompetence, for example, Clinical-Etiology-Anatomy-Pathophysiology classification C5 or C6.^43^
- Contraindication to at least medium compression (18–24 mm Hg).
- Unable to comply with CT.
- Suspected to have a non-KC diagnosis or require further surgery.
- Previously taken part in Healing of ExcisionAl wounds on Lower legs by Secondary intention (Phase 2).

### Recruitment/consent

The trial will recruit participants from SCSCs including district general and teaching hospital settings. The recruitment centres may comprise dermatology surgery and/or plastics surgery services with surgery scheduled in outpatient clinic and day case surgery facilities. Research centres will be required to have obtained local confirmation of capability and capacity, undertake a site initiation meeting with the Clinical Trials Research Unit (CTRU) and receive a CTRU green light prior to the start of recruitment into the trial.

Informed consent will be obtained prior to the participant undergoing procedures that are specifically for the purposes of the study. The right of a participant to refuse participation or withdraw at any time from the study without giving reasons will be respected. Informed consent can be taken either (a) As remote e-consent, (b) Remote verbal consent over the telephone/video calling and (c) Face-to-face during an SCSC visit on a paper form or using an e-consent format. The mode of informed consent used will depend on patient and participating site preference, and their accessibility to a device to support the e-consent process. Patients will be provided with information on the e-consent process to help inform their decision on the method of consent.

For the qualitative sub-study, the target population will be patients randomised to the CT arm of the main trial and who have undergone or are currently undergoing CT. The patients will be those who have opted into the sub-study.

Patients are referred from a healthcare professional to an SCSC, reflecting the variation in referral, diagnosis and surgical scheduling. The referral will usually suggest a potential diagnosis of a KC on the lower leg and be accompanied by photographs. Several pathway options may then be available for identifying eligible participants depending on local arrangements. Identification can occur during telephone/video or face-to-face diagnostic assessment and scheduling of surgery as part of the routine clinical care or where potential trial eligibility (ie, suspected KC on the lower leg and planned HBSI) is observed.

*NB* If a patient consents with the expectation that the wound will be HBSI but surgery is completed with primary closure, then randomisation will not take place. The likelihood of this happening will be explained to the patient during the informed consent process.

### Randomisation

Randomisation will take place immediately postoperatively (in theatre or postoperative recovery area). 396 participants will be randomised in a 1:1 allocation ratio (198 per group) to SC or SC+CT stratifying by:

- Centre.
- Immediate post-excision wound area (6.0 cm^2^ or less/more than 6.0 cm^2^ prior to any partial closure).^23^
- Wound depth (excision to fat/excision to fascia or periosteum).

Randomisation will be performed via a central, independent, secure 24-hour automated randomisation system provided by Leeds CTRU using a minimisation algorithm (including a random element) to ensure allocation concealment.

### Post-randomisation re-excision of wound

In the event that a patient undergoes further surgery on their wound, they will continue in the trial unless the wound is no longer HBSI, for example, primary wound closure or the application of a skin flap or graft. Patients will be clinically withdrawn at this point and no further data collected.

### Blinding

Due to the nature of the interventions, it is not possible to blind participants, but assessments/evaluation will be carried out in a blinded manner.

### Interventions

#### Standard care (SC) alone

The participant will receive the best SC provided by the local recruiting centre. On the day of surgery, excision of the KC is undertaken. The surgeon may perform partial closure (eg, purse string suture) and then the wound is dressed as per local practice. Immediate postoperative dressings may include haemostatic products such as alginate dressings. The patient is discharged with a standard information leaflet which includes contact phone details in case of concern about bleeding, infection or other AE.

Postoperative care and wound management will be provided according to local policies, typically seen twice weekly initially for 2–4 weeks in the SCSC. Subsequently visits reduce in frequency with either secondary care or primary or community care on a planned or ad hoc basis until healed. Additional advice regarding mobility/activity, emollient use and skin care may be provided by the attending clinical teams.

#### SC+CT

SC will be provided as previously described. In addition, CT will be initiated on the day of surgery in the SCSC and continued throughout the episode of wound treatment until healing. Trial CT will deliver pressures between 18–40 mm Hg at the ankle, that is, CT will comprise below-knee hosiery or bandage system.^18^ Bandages will be applied to the lower limb, excluding the toes and finishing below the knee. Hosiery must be a minimum of below-knee and may be open or closed toe. A list of approved compression therapies is available.

Compression choice (hosiery vs bandage system) will be based on patient preferences and clinician’s discretion from research-informed^13 14 18^ trial-approved product list; the rationale for choice will be reported, for example, ability to self-manage, compression-related discomfort, patient lifestyle, amount of oedema, pain, frequency of dressing changes, planned follow-up pathway. The underlying principle is the provision of compression until healing.

Where randomised to CT, compression will be applied as soon as possible, and additional information provided in respect of the CT. Frequency of hosiery change will be determined by ability to self-care and/or frequency of SC wound dressing changes. Frequency of bandage system changes will be determined by frequency of SC wound dressing changes.

### Assessments and data collection

Only data items that meet the study objectives have been included. PPIE involvement in the design of patient information and data collection forms and participant feedback has been incorporated to promote data completion and minimise missing data.

Baseline assessments and follow-up until confirmed healing (weekly up to 26 weeks then monthly where applicable, maximum 52 weeks) will be undertaken by a member of the clinical research team (eg, clinician, clinical research nurse or Registered Healthcare Professional (RHCP)). Follow-up phone calls may be completed by a non-RHCP, for example, clinical trials assistant.

Follow-up data collection will be via phone calls or can be face-to-face if attending the SCSC for routine clinic visits. If this is not possible, a member of the clinical research team may visit the patient in a community wounds clinic or at home. In-person visits are required at week four and confirmation of healing. Where an in-person visit with a member of the clinical research team is not possible, then a video call using NHS-approved technology with a photograph taken via screenshot can be submitted. In the unlikely event that neither an in-person visit nor a video call using NHS-approved technology can take place, then a photograph taken by the participant or participant’s associate can be submitted to the local research team who will then send by Secure File Transfer to CTRU.

Participants recruited more than 6 months before the end of the recruitment phase will be followed up to 4 weeks after confirmed healing, up to a maximum of 52 weeks (+7 days). Participants recruited during the final 6 months of the recruitment phase will be followed up to 4 weeks post confirmed healing for between 26 and 52 weeks (+7 days).

When the participant indicates their wound has healed at a follow-up visit or phone call, a confirmation of healing visit will be arranged. They will be asked to remove all dressings and stop using compression prior to the confirmation visit. A blinded assessor (healthcare professional) will assess and photograph the wound. If confirmed, a follow-up phone call will be completed 4 weeks after the healing confirmation visit (up to a maximum of 52 weeks post randomisation) to determine whether the wound has broken down.

If the wound is assessed as unhealed at the confirmation of healing visit, a photograph will be taken, and routine weekly/monthly phone calls and randomised treatment will be reinstated.

Medical record review will be undertaken at 26 and 52 weeks (where applicable, if not healed at 26 weeks) to capture related SAEs (including hospitalisations and related infections) and date patient last known to have an unhealed wound. Record review to also be performed on an ad-hoc basis if the participant dies, withdraws or is lost to follow-up.

The following information will be recorded:

#### Baseline assessment on the day of surgery/randomisation

- Demographic information.
- Relevant medical history and clinical assessments.
  - ABPI or Toe Pressure.
  - CEAP.
  - Factors affecting healing.
  - Mobility.
- Questionnaire.
  - EQ-5D-5L.
- In theatre.
  - Post excision wound measurement – length, width, depth (excision to fat/excision to fascia or periosteum).
  - Type of partial closure (purse string suture, side-to-side partial closure, eg, with pulley sutures or buried sutures, none).
  - Post partial closure measurement (where applicable) – length and width.
- Following randomisation
  - Participant trial ID number.
  - Randomisation allocation and details of intervention application.
  - Date.
  - Antibiotics.
  - Related expected adverse and SAEs.

#### Follow-up phone calls/clinic visit

- Wound status healed/unhealed.
- Antibiotic use.
- Any further surgery on the wound.
- Treatment application and adherence.
- Related expected/unexpected adverse and SAEs.

#### Week 4 clinic visit*

- Wound assessment healed/unhealed.
- Wound measurement.
- Antibiotic use.
- Any further surgery on the wound.
- Treatment application and adherence.
- Photography.
- Related expected adverse and SAEs.
- Questionnaires.
  - Modified Bluebelle WHQ.
  - EQ-5D-5L.
  - Health Resource Use.
  - Intervention acceptability (CT only).

*If the week 4 clinic visit does not take place, a follow-up phone call will be made and questionnaires will be completed remotely.

#### Week 12 postal/electronic data collection

- Questionnaires.
  - Modified Bluebelle WHQ (if unhealed).
  - EQ-5D-5L.
  - Health Resource Use.

#### Week 26 postal/electronic data collection and record review

- Postal/electronic questionnaires.
  - Modified Bluebelle WHQ (if unhealed).
  - EQ-5D-5L.
  - Health Resource Use.
- Record review: a member of the research team will complete a review of the participant medical records and record the following information:
  - Related and expected SAEs.
  - Date wound last known to be unhealed (if applicable).
  - Histology results/diagnosis.

#### Week 52 record review (if wound not healed at week 26)

- A member of the research team will complete a review of the participant medical records and record the following information *only for participants whose wounds had not healed by Week 26*:
  - Related and expected SAEs.
  - Date wound last known to be unhealed (if applicable).
  - Histology results/diagnosis.

#### Week 52 postal/electronic data collection (if wound not healed at week 52)

- Postal/electronic questionnaires.
  - Modified Bluebelle WHQ.
  - EQ-5D-5L.
  - Health Resource Use.

An ad hoc record review will be carried out if patient dies, withdraws or is lost to follow-up.

### Assessment of healing

Healing is defined as complete epithelial cover in the absence of a scab, eschar or crust (adapted from Chetter *et al*^20^ and Arundel *et al*^24^). Where participants indicate their wound has healed during a follow-up phone call, a confirmation of healing visit will be arranged within 7 days.

### Questionnaires

Participants must provide consent to complete questionnaires at the point of joining the study but may decline to complete a questionnaire at any time during follow-up and remain in the study. Baseline, week 4 and blinded healing assessment visit questionnaires will be administered in clinic on paper/verbally. If the participant does not attend the in-person week 4 clinic visit then questionnaires can be administered over the phone. Participants can choose their preferred method to receive other follow-up questionnaires (electronic/paper). Participants will receive 1 reminder message via their preferred method if a completed questionnaire has not been received within the expected time frame (2 weeks for return by post and 1 week for electronic submissions). The following questionnaires will be administered during the study:

- Modified Bluebelle WHQ.
- POSAS.
- Intervention acceptability (CT only).
- EQ-5D-5L.
- Healthcare Resource Use.

#### Modified bluebelle wound healing questionnaire (WHQ)

The modified Bluebelle WHQ^20^ has been adapted from the original Bluebelle WHQ^25^ for relevance for patients with open wounds by removing three items relating to spontaneous or deliberate wound dehiscence and use of dressings given these would not be relevant in this population. The time frame for the questionnaire has also been adapted to indicate that the questionnaire should be completed since the participant had their open wound (at wound healing or since the last modified Bluebelle questionnaire was completed – weeks 4, 12, 26 and 52 weeks (where applicable, if wound is not healed)) post randomisation and at healing, where applicable rather than the time since hospital discharge.

#### Patient and observer scar assessment scale (POSAS)

The POSAS^21^ measures scar quality by evaluating visual, tactile and sensory characteristics of the scar from the perspective of the observer and patients.

#### Intervention acceptability

Intervention acceptability will be explored through questions developed with PPI input to consider comfort, convenience and appearance. Details will be completed by participants in the intervention arm at week 4 and at the confirmation of healing visit.

#### EQ-5D-5L

EQ-5D-5L questionnaires will be completed face to face at baseline and week 4. Postal/electronic versions will be administered at weeks 12 and 26 and at week 52 (where applicable, if wound is not healed). EQ-5D-5L is an accepted, five-item, generic, health-related quality of life measure including five items that can be combined to provide a single assessment of utility of life in a particular health state.^26^ The EQ-5D is a generic instrument (www.euroqol.org) and forms part of the National Institute for Health and Clinical Excellence (NICE) reference case for cost per QALY analysis.

#### Health resource use

Health resource use questionnaires will be completed at 4, 12 and 26 weeks and at week 52 (where applicable, if wound is not healed).

### Sample size

The sample size estimates are based on HEALS feasibility cohort data on time to healing and clinical and health economic considerations regarding an important reduction in number of days until complete healing. Assuming a median time to healing of 81 days in the SC arm^12^, a total of 396 randomised participants (198 per group) will provide 90% power for detecting a target effect size of 30% reduction in time to healing in the SC+CT group (57 days, HR=1.43), 2-sided log-rank test at 5% significance level and 10% attrition.

For the qualitative sub-study, it is expected that up to 25 patients will be identified to achieve a demographic spread and to achieve adequate depth and variety of data to sufficiently answer the study questions. We would aim for 1–2 patients from each recruitment site with representation from diverse sex and age groups.

### Statistical analysis

The statistical analysis will follow a predetermined, approved, version-controlled statistical analysis plan (SAP), and reporting will be in line with Consolidated Standards of Reporting Trials (CONSORT) standards.^27^ Statistical monitoring of safety data and underlying assumptions of the statistical design will be conducted and reported to the data monitoring and ethics committee (DMEC) according to an agreed DMEC Charter.

Baseline demographic data will be summarised to assess comparability between treatment arms using means and SD or medians and IQRs as appropriate for continuous variables, and numbers and percentages for binary and categorical variables. These same methods will be used to generate descriptive statistics where required.

#### Primary analysis of primary outcome measure

Primary analysis of the primary outcome measure, time to complete healing will be conducted on the intention-to-treat population using a multivariable Cox Proportional Hazards (PH) regression model adjusted for the minimisation factors post-excision wound area and wound depth; the potential for fitting centre as a random effect will be explored. Re-excision will be considered as a time-dependent covariate. Estimated HR will be presented with 95% CI and significance. Unadjusted Kaplan-Meier plots and estimates of survival functions (where ‘survival’ corresponds to *healing not observed*) and the stratified log-rank test statistic and its significance will be also reported. Should the PH assumption appear to be violated, an appropriate alternative analysis method will be used.

#### Sensitivity analyses

- *Death as a competing risk*: should incidence of death be substantial as a competing risk event, a multivariable Fine & Grey regression model^28^ adjusted for the minimisation factors may be fitted to the primary outcome measure.
- *Treatment adherence*: patterns of treatment adherence will be explored, and an appropriate statistical method taking adherence into account while respecting the randomisation (eg, rank preserving structural or structural nested failure time model^29–31^ or marginal structural model)^32 33^ will be used to model causal treatment effect for observed adherence.
- *Alignment with Health Economic analysis*: a parametric model consistent with that which is required in the health economic analyses (see Economic Evaluation section) for providing transition probabilities beyond the time frame of the trial will be fitted to the primary outcome measure.

#### Analysis of secondary outcome measures

- *Wound infection measured by modified Bluebelle (WHQ*): a multivariable, random coefficients, repeated measures linear regression model will be fitted to modified Bluebelle WHQ score over time (until complete wound healing), including minimisation factors and randomised treatment. Centre, participant and participant-by-time interaction random effects will be explored. Time, treatment and treatment-by-time interaction will be fitted as fixed effects. An estimate for the magnitude of the treatment-by-time interaction and corresponding 2-sided 95% CI will be reported.
- *Number of days prescribed antibiotics for infection of the wound*: a multivariable Poisson regression model will be fitted to whether participant has been prescribed antibiotics over time, with an offset term for time at risk of being on antibiotics, including the minimisation factors and randomised treatment. Centre and participant random effects will be explored. An estimate for the difference in number of days where antibiotic prescribed and 95% CI will be reported.
- *Scar Quality (POSAS*): a multivariable linear regression model will be fitted to both participant and clinician POSAS scores, including the minimisation factors and randomised treatment. Centre random effects will be explored. Estimates for the differences in POSAS scores between the SC and SC+CT arms and 95% CIs will be reported.
- *Safety*: events including related complications and hospitalisations to healing (maximum 52 weeks post randomisation) will be summarised descriptively by treatment received.
- *Associations between partial closure method, post partial closure wound area and time to healing*: partial wound closure method and post-partial closure wound area will be included as additional covariates in the primary endpoint analysis model as described above. Wound area at baseline and 4 weeks post randomisation will be calculated using the elliptical method^34^ from maximum length and width measurements provided by site; time to complete wound healing will be determined as per the primary endpoint measure.

#### Exploratory analyses

- *Development of a predictive marker of healing*: a multivariable model to predict time to complete wound healing incorporating the minimisation factors and Week 4 measurements of reduction in wound area from baseline will be developed in line with the Prognosis Research Strategy (PROGRESS) initiative.^35–38^
- *Intervention acceptability and treatment adherence*: both will be summarised descriptively overall, and treatment adherence will also be summarised by randomised treatment group.
- *Post wound healing breakdown*: descriptive summaries including proportions of index wound status 1 month post healing confirmation (wound healing maintained or wound breakdown) by randomised treatment group and overall will be presented.

#### Missing data considerations

The extent of missing data for each primary and secondary endpoint will be presented by trial arm along with reasons where available. Missing data strategies appropriate for each primary and secondary analysis will be employed. For example, should primary endpoint censoring appear informative, inverse probability of censoring weighting may be used. Further details on how missing data will be handled in each analysis are included in the SAP.

### Economic evaluation

The economic evaluation will assess the cost-utility of SC+CT compared to SC alone using within-trial analysis and a DAM to assess the argument over a longer time perspective. The proposed methods for the economic evaluation will follow the reference case set out by NICE.^39^

The within-trial analysis will be undertaken to estimate the resource use, costs and effects for both comparator arms for the 26-week follow-up period. The DAM will be developed over the course of the project, extrapolating to 52 weeks to help in assessing alignment with and validation by the within-trial analysis and providing some insight on cost-effectiveness drivers. The DAM will be based on a Markov model with states based on healed, unhealed, infected and dead. An NHS and Personal Social Services perspective for costs will be adopted.

Health resource use data will be collected from a questionnaire (see Data Collection and Questionnaire section) and from trial case report forms (CRFs). The information will include primary, secondary and community resource use. Unit cost data will be obtained from national databases such as the *National Schedule of Costs* (NHS England), the *Personal Social Services Research Unit (PSSRU) Costs of Health and Social Care* and the *British National Formulary (BNF*). All resource use will be valued in monetary terms using appropriate UK unit costs with the price year for which all costs can be valued and required adjustments made for inflation using the Office of National Statistics (ONS) Gross Domestic Product (GDP) deflator index.

Treatment costs include the costs of CT and of SC post-op care and wound management (clinic visits, dressings provided, staff time). Participants’ responses to the EQ-5D-5L questionnaire will be converted to utility scores using the newly published UK value set for the 5L version.^40^

Results of the primary economic analysis will be presented as incremental cost-effectiveness ratios (ICERs) for each arm expressed in terms of QALYs. Incremental net monetary benefit and incremental net health benefit statistics will also be computed. NICE considers a cost per QALY within the range of £20 000–£30 000 to be acceptable. The approach to missing data will follow good practice guidelines for cost-effectiveness analysis alongside clinical trials.^41^ Analytical techniques will be jointly determined with the statistical co-applicants to ensure consistency across the project (eg, multiple imputation techniques). Deterministic and probabilistic sensitivity analyses will be used to assess the impact of uncertainty on the results. A health economic analysis plan containing details of the economic evaluation to be conducted will be prepared before the completion of data collection.

### Qualitative sub-study

The qualitative research sub-study will be in the form of virtual semi-structured interviews. These will be audio recorded, transcribed and analysed.

#### Data collection

Up to 25 patients will be identified to achieve a demographic spread and to achieve data adequacy, that is, adequate depth and variety of data to sufficiently answer the study questions. We aim to have representation from most of our recruiting centres with a spread of demographics. We will use a purposeful sampling framework to include those who have found the CT to be acceptable and unacceptable, using specific characteristics (see table 1 below).

**Table 1:** Purposeful sampling characteristics guide

Purposeful sampling factors		Minimum number of patients
	Functional status	
Patients	Self-caring (applies CT themselves) Needs care (needs assistance with CT)	3 3
	Age	
	<65 years ≥65 years	4 4
	Sex	
	Male Female	5 5
	Prior experience with CT	
	Yes No	3 3
	Type of CT	
	Bandaging Compression hosiery	3 3
	Duration of CT	
	Undergoing CT <6 weeks Undergoing CT >6 weeks	2 2
	Completion of CT	
	Full completion Ongoing active therapy Discontinued	2 2 2
	Experience with CT	
Hospitals	Routinely provides CT prior to trial Does not routinely provide CT prior to trial	3 3

The interviews will be guided by a topic guide covering various aspects related to CT, including effectiveness, comfort, challenges, impact on daily activities, pain levels, duration of treatment, satisfaction with healthcare support and factors influencing treatment continuation. The topic guide is developed based on our previous research and informed by the responses to the Intervention Acceptability Questionnaire administered 4 weeks after CT allocation and discussions with members of the trial team, with PPI.

Interviews will be recorded and transcribed verbatim using a University of Leeds approved transcription service. Data will be subject to thematic analysis of the qualitative data obtained from the interviews, guided by The Framework Method.^42^ An iterative-inductive approach will be employed to generate emergent categories and ideas based on specific observations on patient experiences rather than prior concepts, ensuring diversity and complexity in the analysis. Additionally, we aim to respect individual cases while identifying comparative themes and patterns. The transcripts will be coded into themes and sub-themes to identify patterns, common experiences and key findings. Iterative analysis will allow for the emergence of new themes while refining the coding framework.

### Data management

All information collected during the trial will be kept strictly confidential. Information will be held securely on paper and electronically at the Leeds CTRU. The CTRU will comply with all aspects of the 2018 Data Protection Act and operationally this will include:

- Consent from participants to record personal details including name*, date of birth*, address and telephone number, email address, NHS or CHI number, hospital number, GP name and address.
- Appropriate storage, restricted access and disposal arrangements for participant personal and clinical details.
- Consent from participants for access to their medical records by responsible individuals from the research staff or from regulatory authorities, where it is relevant to trial participation.
- Consent from participants for the data collected for the trial to be used to evaluate safety and develop new research.
- Participant name, address, email address and telephone number will be collected when a participant is randomised into the trial but all other data collection forms that are transferred to or from the CTRU will be coded with a trial number and will include two participant identifiers, usually the participant’s initials and date of birth.
- Where central monitoring of source documents by CTRU (or copies of source documents) is required (such as scans or local blood results), the participant’s name must be obliterated by site before sending.
- Where anonymisation of documentation is required, sites are responsible for ensuring only the instructed identifiers are present before sending to CTRU.

If a participant withdraws consent from further trial treatment and/or further collection of data their data will remain on file and will be included in the final study analysis.

### Data monitoring

Data will be routinely monitored for quality and completeness by the CTRU. Missing data items are automatically flagged. Targeted strategies for identifying compliance issues and obtaining these data items will be undertaken, including monitoring data return progress, escalation where required and proactively offering assistance and support to research teams at sites. Data will be chased until it is received, confirmed as not available or the trial is at analysis. However missing data items will not be chased from participants (although missing questionnaires sometimes are). The (CTRU/Sponsor) will reserve the right to intermittently conduct source data verification exercises on a sample of participants, which will be carried out by staff from the (CTRU/Sponsor). Source data verification will involve direct access to patient notes at the participating hospital sites and the ongoing central collection of copies of consent forms and other relevant investigation reports.

## Ethics and dissemination

### Ethics

The study has been approved by the Yorkshire & The Humber – Leeds East Research Ethics Committee (ref 23/YH/0247).

#### Oversight/trial monitoring group (TMG)

The TMG comprising of the Chief Investigators, CTRU team and co-applicants, will be assigned responsibility for the clinical set up, ongoing management, promotion of the trial and for the interpretation and publishing of the results. Specifically the TMG will be responsible for (1) Protocol completion, (2) CRF development, (3) Obtaining approval from the Main REC and supporting applications for Site Specific Assessments, (4) Completing cost estimates on project initiation, (5) Nominating members and facilitating the TSC and DMEC, (6) Reporting of SAEs, (7) Monitor of screening, recruitment, treatment and follow up procedures, (8) Auditing consent procedures, data collection, trial end point validation and database development and (9) Central review of photographs.

#### Trial steering committee (TSC)

The TSC will provide overall supervision of the trial, in particular trial progress, adherence to protocol, participant safety and consideration of new information. It will include an independent chair, not less than two other independent members and a consumer representative (PPI). The Chief Investigators and other members of the TMG may attend the TSC meetings and present and report progress. The Committee will meet 6 monthly.

#### Data monitoring and ethics committee (DMEC)

An independent DMEC will review the safety and ethics of the trial by reviewing interim data during recruitment. The DMEC will be provided with detailed unblinded reports prepared by the CTRU at regular intervals containing the information agreed in the data monitoring analysis plan. The Committee will meet annually as a minimum.

A Trial Monitoring Plan will be developed and agreed by the TMG and TSC based on the trial risk assessment. This may include on site monitoring.

### Dissemination

The main results of the trial will be published as open-access in the NIHR library and in high-impact peer-reviewed journals.

## Patient and public involvement

The trial was supported by patient representatives from related charities and previous PPIE groups and were involved in the set-up of the trial. In particular, our UKDCTN-funded focus group comprised eight patients with previous experience of lower limb excision of KCs. We discussed their experiences and exchanged ideas about important outcomes. They felt it was acceptable to be randomised to either SC or SC+CT. We discussed the follow-up option of weekly contacts, and the group felt this was acceptable. This informed the data collection schedule. They felt capturing information about complications such as infection, the time the wounds took to heal and the appearance of scar/healed wound were important, hence relevant data will be collected. Our PPI group also felt that, given the choice, most would prefer to wear hosiery than bandages. They also felt hosiery increased opportunities for self-care.

The trial has PPI representatives on the TMG and TSC who have provided input into the patient information sheet and other trial documentation intended for use by patients. The PPI representatives also provide input into the design and conduct of the trial at the regular meetings.

This high-level involvement in project management aims to ensure patients’ perspectives are fully integrated in key decisions about the trial, delivery and interpretation/dissemination of findings.

## Discussion

The incidence of KCs is increasing due to an ageing population and social change including elevated levels of sun exposure. Subsequently, there is a growing use of CT in clinical practice without robust evidence of clinical- and cost-effectiveness. Given this rise in use, a high-quality RCT is required. HEALS2 will aim to generate the evidence needed to identify the relative effectiveness of CT in this population.

### Trial status

Recruitment started in February 2024 and will be completed by the end of August 2026 with a 6-month follow-up period. As of 31 December 2025, 276 participants have been randomised. The trial is expected to be completed by 31 December 2026. The full trial protocol is available on the National Institute for Health Research journals library https://fundingawards.nihr.ac.uk/award/NIHR151863.
