# Supplementary material for: Pragmatic randomised controlled trial to evaluate the clinical and cost-effectiveness of compression therapy for surgical wounds healing by secondary intention following excision of lower limb keratinocyte cancers in UK skin cancer surgery centres (HEALS2 trial): study protocol
Source: BMJ Open. 2026 Sep 7;16(9):e115191. doi: 10.1136/bmjopen-2025-115191 (PMC13560984; doi:10.1136/bmjopen-2025-115191)
Supplement: Supplementary file 1 [file bmjopen-2025-115191.reviewer_comments.pdf]

PEER REVIEW HISTORY

BMJ Open publishes all reviews undertaken for accepted manuscripts. Reviewers are asked to complete a checklist review form and are provided with free text boxes to elaborate on their assessment. These free text comments are reproduced below.

ARTICLE DETAILS

Title (Provisional)

A pragmatic Randomised Controlled Trial to evaluate the clinical and cost effectiveness of Compression Therapy in the healing of surgical wounds Healing by Secondary Intention following excision of lower limb Keratinocyte Cancers, in UK skin cancer surgery centres (HEALS2 trial): study protocol

Authors

Konwea, Henrietta; Ransom, Myka; Abbott, Rachel; Ashraf, Iqra; Bojke, Chris; Brown, Sarah Tess; Collier, Howard; Connor, Joanna; Gilberts, Rachael Mary; Mann, Jasmine; Muli, Annah; Nixon, Jane; Pynn, Emma; Veitch, David; Wernham, A. G. H.; McGinnis, Elizabeth

---

VERSION 1 - REVIEW

Reviewer 1

Name Epstein, David

Affiliation Universidad de Granada - Campus de Cartuja, Department of Applied Economics

Date 02-Apr-2026

COI None

I am a health economist.

The study appears to measure key safety, re-hospitalizations and QOL measures only for 6 months or until healing, while for unhealed patients, these measures will be collected up to one year.

I strongly advise the trialists to reconsider this. I recommend that follow up for these key outcomes (safety, hospitalizations, resource use and EQ5D) are continued in all patients until the trial close (one year).

I could not see exposition of the arguments for truncating collection of these outcomes. Is it to minimize the burden on participants? However, the EQ5D is a very simple questionnaire, and can be collected from the patient online or by telephone. Likewise . data collection on major complications or hospitalziations related to the disease or treatments after healing will not be a burden for patients. I agree that a full health economic resource use

questionnaire might not be necessary after healing. But follow up using simple, quick questions is still feasible. The trial has a primary outcome which is healing, but secondary outcomes are also important.

If you do not collect QOL in patients after 26w in patients who have healed, then, by definition, you will not be able to demonstrate that compression improves QOL.

Likewise, if you do not collect data after healing, you are assuming, rather than demonstrating, that there are no further events. The wound may recur, or complications may take time to materialize. The benefits of data collection hugely outwieght the disadvantages for patients and the cost for the trialists.

Within -trial analysis for health economics for 26 weeks is insufficient. There may be many unhealed wounds at this time. It is also inefficient . You have 52 weeks follow up in some patients and are not using all these data. If there are few health economic data from the trial in healed patients, this will result in a major limitation for the construction of the decision analytic model (as you will not know the QOL in these patients). The within trial analysis would be better if conducted for 52 weeks , and the DAM to extrapolate for longer term outcomes (e.g. if there are unhealed patients at 52 weeks)

**Reviewer**	**2**
**Name**	**Kelson, Zoe**
**Affiliation**	**University of Exeter, Mathematics**
**Date**	**20-May-2026**
**COI**	**None**

The aim of this RCT is to evaluate the clinical and cost-effectiveness of SC + CT in the healing of surgical wounds HBSI following excision of lower limb KCs.

Reviewer commemt:

"The study protocol complies with the Standard Protocol Items: Recommendations for Interventional Trials (SPIRIT) Statement."

Thanks to the authors for providing a copy of the SPIRIT checklist.

"HEALS2 is a multicentre, prospective, Phase III, parallel group, open-label, randomised controlled trial with embedded internal pilot and blinded endpoint assessment." [Abstract]

and

"HEALS2 is a multicentre, prospective, Phase III, parallel group, open-label, randomised, controlled trial (RCT) with embedded internal pilot, blinded endpoint assessment, economic evaluation, minimum 26- week/maximum 52-week follow-up, comparing time from randomisation to complete surgical wound healing (epithelialisation) by secondary intention

between control (SC) and intervention (SC + CT) groups. The qualitative sub-study entails virtual semi-structured interviews with participants randomised to CT. These will be audio-recorded, transcribed and analysed. "

A rigorous study design is proposed by the authors.

The authors clearly detail the planned primary, secondary and exploratory objectives, as well as describe the qualitative substudy.

"All adult patients attending a UK secondary care skin cancer surgery centre (SCSC) will be screened for suitability according to the eligibility criteria (Box 1), by the attending clinical/research team if they have a suspected KC on the lower leg which is planned to be excised and HBSI...

The trial will recruit participants from SCSCs including district general and teaching hospital settings."

Can the authors please comment on how they intend to demonstrate the representativeness of the study sample?

"Randomisation will take place immediately post-operatively (in theatre or post-operative recovery area). 396 participants will be randomised in a 1:1 allocation ratio (198 per group) to SC or SC + CT stratifying by: • Centre • Immediate post-excision wound area (6.0cm 2 or less/more than 6.0cm2 prior to any partial closure) [24] • Wound depth (excision to fat/excision to fascia or periosteum)"

The authors clearly describe the randomisation process, noting the stratification variables that will be used in the allocation.

"Baseline assessments and follow-up until confirmed healing (weekly up to 26 weeks then monthly where applicable, maximum 52 weeks) will be undertaken by a member of the clinical research team (e.g. clinician, clinical research nurse or Registered Healthcare professional (RHCP))."

and

"Missing data will be chased until it is received, confirmed as not available or the trial is at analysis. However missing data items will not be chased from participants (although missing questionnaires sometimes are)."

Can the authors please comment on how missing data be minimised?

"The extent of missing data for each primary and secondary endpoint will be presented by trial arm along with reasons where available. Details on how missing data will be handled in each analysis is included in the SAP."

Can the authors please provide a description of how they intend to handle missing data in the analysis?

"Assuming a median time to healing of 81 days in the SC arm(12) a total of 396 randomised participants (198 per group) will provide 90% power for detecting a target effect size of 30% reduction in time to healing in the SC + CT group (57 days, HR =1.43), 2-sided log-rank test at 5% significance level and 10% attrition"

The authors have satisfactorily communicated the basis for the a priori sample size calculation and associated assessment of study power.

"The statistical analysis will follow a predetermined, approved, version-controlled statistical analysis plan (SAP), and reporting will be in line with Consolidated Standards Of Reporting Trials (CONSORT) standards"

The authors demonstrate that rigor will be applied in the development of the statistical development plan (SAP).

"Primary endpoint analysis will be conducted on the intention-to-treat (ITT) population" [Abstract]

and

"Primary analysis of the primary outcome measure, time to complete healing will be conducted on the intention-to-treat (ITT) population"

Can the authors also consider undertaking a per protocol (PP) sensitivity analysis?

"...using a multivariable Cox Proportional Hazards modelling.

Secondary outcomes and safety will be evaluated using other regression models and descriptive statistics, respectively. Cost-effectiveness will be evaluated using within-trial analysis and decision-analytic modelling" [Abstract]

and

"using a multivariable Cox Proportional Hazards (PH) regression model adjusted for the minimisation factors post-excision wound area and wound depth; the potential for fitting centre as a random effect will be explored. Re-excision will be considered as a timedependent covariate. Estimated HR will be presented with 95% CI and significance. Unadjusted Kaplan-Meier plots and estimates of survival functions (where 'survival' corresponds to healing not observed) and the stratified log-rank test statistic and its significance will be also reported. Should the PH assumption appear to be violated, an appropriate alternative analysis method will be used."

Appropriate modelling methods are planned, suitbly adjusting for the stratification variables.

"Sensitivity analyses"

A number of insightful sensitivity analyses are pre-specified by the authors.

"Trial status

Recruitment started in February 2024 and is expected to complete by December 2025 with a 6-month follow-up period. As of 31st August 2025, 215 participants have been randomised. The full trial protocol is available on the National Institute for Health Research journals library https://fundingawards.nihr.ac.uk/award/NIHR151863"

Can start and expected end dates please also be noted by the authors in the Abstract and Methods?

"Limited to patients with secondary intention healing (excludes those who have primary closure, flap or graft) • Limited to those who have a suspected KC as non-KC e.g. melanoma usually experience a different clinical pathway" [Strengths and Limitations of this study]

The authors acknowledge the study limitations in the protocol.

VERSION 1 - AUTHOR RESPONSE

REVIEWER COMMENT	AUTHORS' RESPONSE
1. I am a health economist.<br>The study appears to measure key safety, re-hospitalizations and QOL measures only for 6 months or until healing, while for unhealed patients, these measures will be collected up to one year. I strongly advise the trialists to reconsider this.<br><br>I recommend that follow up for these key outcomes (safety, hospitalizations, resource use and EQ5D) are continued in all patients until the trial close (one year).	**Action: No change to manuscript**<br><br>We understand where the health economist is coming from as this is an unusually designed trial with uneven follow-up. As health economists ourselves our own preferences would be for complete coverage of all data collection for 52 weeks, but this is not an option. The duration of the complete data collection was indeed a much discussed and considered issue and the arguments agreed with funders – revising this is not possible. And so the proposed analysis is based on what we think is optimal given the actual trial that has been funded.<br><br>Thus from our perspective we regard the HEALS2 trial as a 6-month trial, for which there will be full (weekly) data collection for all patients healed or not. There is then this option to collect non-random data on

	selected non-healed patients which we can use to check modelled results rather than feature within the model construct itself.<br><br>From that perspective the options we have are to:<br><br>a) build a 6-month within-trial and a longer-term decision analytical model (DAM) based on extrapolations from the 6-month data and check to see if the additional data is consistent with the modelled data.<br>b) build a 6-month within-trial and a longer-term DAM based on extrapolations from the 6-month data and ignore the additional data.<br>c) build a 12- month within-trial and a longer-term DAM based on extrapolations from the 12-month data and try to impute the non-randomly missing data.<br><br>There is no possibility to revise the trial to have an option d which is to extend the regular data collection to 12 months. The key issue is whether 6 months regular data is sufficient to build a model, and we think it clearly is making options a and b superior to c.<br><br>Importantly, we considered that wounds from the

	excision of KC are acute wounds and are neither diabetic foot nor venous ulcers; clinical evidence with expert opinion suggests that most of the KC wounds heal within 6 months, following surgery:<br><br>• Findings from a preceding systematic literature review identified only 2 cohort studies with reported mean time to healing for no compression (n=53) of 81 days and compression (n=10(14) of 50 days. Please note that no RCTs for our study population was identified in this review.<br>• Unpublished local audit data at a participating centre of the trial also reported that 88% (22/25) of patients with compression healed within 6 weeks.<br><br>Thus the trial length is relatively generous and longer than is usually observed within this field. We strongly reject any suggestion that 6 months is too short a period on which to build an economic model.<br><br>The follow-up of a likely very small group of unhealed is of additional interest to the HEALS2 Study Group but should not be regarded as a truncation. We are not using this data to build counterfactual arguments but instead will look to see whether modelled extrapolations appear consistent with observed

	outcomes for the unhealed wounds.
2. I could not see exposition of the arguments for truncating collection of these outcomes. Is it to minimize the burden on participants?<br><br>However, the EQ5D is a very simple questionnaire, and can be collected from the patient online or by telephone. Likewise. data collection on major complications or hospitalizations related to the disease or treatments after healing will not be a burden for patients. I agree that a full health economic resource use questionnaire might not be necessary after healing. But follow up using simple, quick questions is still feasible. The trial has a primary outcome which is healing, but secondary outcomes are also important.	**Action: No change to manuscript**<br><br>Please see response above to reviewer comment #4. Given the above response there is no truncation, the health economics is built on the complete 6-month data collection.
3. If you do not collect QOL in patients after 26w in patients who have healed, then, by definition, you will not be able to demonstrate that compression improves QOL.	**Action: No change to manuscript**<br><br>This suggests that we can never rely on DAMs but will always have to collect data till the last event. We will build and assess a 6-month within trial model and by definition we will be clear about the 6-month limitation of the analysis. We will build a DAM based on 6-month data to extrapolate beyond this period. i.e. we will adopt the standard methods for assessing the argument beyond the constraints of the trial.
4. Likewise, if you do not collect data after healing, you are assuming, rather than demonstrating, that there are no further events. The wound may recur, or complications may take time to materialize. The benefits of data collection hugely outwieght the disadvantages for patients and the cost for the trialists.	**Action:** No change to manuscript<br><br>These wounds are not due to diabetes nor venous issues and in clinical In practice, once the wound is healed, there is no clinical follow-up. We are not aware of any reports of recurrence of surgical wound or complications of management that have tangible economic consequences beyond the 6-

	month period which warrant an economic evaluation. We therefore very strongly dispute that the costs of data collection outweigh an expected negligible benefit of modelling events clinicians do not expect to occur. We are happy to make this assumption clear in the modelling.
5. within -trial analysis for health economics for 26 weeks is insufficient. There may be many unhealed wounds at this time.	**Action: No change to manuscript**<br><br>This is another very strong statement which we fundamentally reject. Given the rates of healing which we observe for such wounds it is clear that a 6-month model is more than sufficient and goes beyond the majority of the literature. Indeed a 52-week within model would most likely be inefficient by increasing costs with very little additional information gain and a full 12-month follow-up would not have been commissioned.
6. It is also inefficient . You have 52 weeks follow up in some patients and are not using all these data. If there are few health economic data from the trial in healed patients, this will result in a major limitation for the construction of the decision analytic model (as you will not know the QOL in these patients). The within trial analysis would be better if conducted for 52 weeks , and the DAM to extrapolate for longer term outcomes (e.g. if there are unhealed patients at 52 weeks)	**Action: No change to manuscript**<br><br>Given the nature of the wounds a 26-week period has been deemed entirely appropriate from a clinical perspective and we expect a very high proportion of wounds to be healed in this period. This is more than sufficient for an economic model. As is completely standard we will use a DAM (using only 6-month data) to extrapolate to longer time periods. There is every indication that the 6-month healing rates will be more than adequate to model beyond that time period – we

	do not believe that is will be a major limitation at all.<br><br>If this had been a standard 26-week trial without the non-random follow up I'm not sure we would have seen any of these objections at all. 6 months is a very generous evidence base for these wounds.<br><br>Unfortunately the addition of a non-random follow-up has tantalisingly dangled the notion of a 52 week within-trial but only if the data collection could be extended such that it was complete throughout – alas this was never a fundable option. And because of the risk of bias trying to build a 52 week within model brings with the current data, we believe treating this as a 26 week within model, informing a longer-term DAM and with the extra data there to compare observed and predicted is the best use of the data. And we strongly defend the position that a 26- week within model is more than enough to establish the economic argument and indeed much more efficient than increasing the trial costs for 52 weeks or even beyond.
7. "All adult patients attending a UK secondary care skin cancer surgery centre (SCSC) will be screened for suitability according to the eligibility criteria (Box 1), by the attending clinical/research team if they have a suspected KC on the lower leg which is planned to be excised and HBSI…<br>The trial will recruit participants from SCSCs including district general and teaching hospital settings."<br>Can the authors please comment on how they intend to demonstrate the representativeness of the study sample?	**Action: Text amended in manuscript**<br><br>Study sites are collecting screening data on patients who are eligible but not recruited. The demographics will be compared with the recruited sample of patients.

8. "Baseline assessments and follow-up until confirmed healing (weekly up to 26 weeks then monthly where applicable, maximum 52 weeks) will be undertaken by a member of the clinical research team (e.g. clinician, clinical research nurse or Registered Healthcare professional (RHCP))."<br>and<br>"Missing data will be chased until it is received, confirmed as not available or the trial is at analysis. However missing data items will not be chased from participants (although missing questionnaires sometimes are)."<br>Can the authors please comment on how missing data be minimised?	**Action: Text amended in manuscript**<br><br>To minimise missing data, data collection was co-designed with PPI representatives, to be efficient and easy to complete for research staff and participants.<br><br>Data compliance is constantly centrally monitored. Missing data is automatically flagged, and sites are contacted asking for the specific data. Where data is not forthcoming, this is escalated, and specific support is given to sites to facilitate data returns.
9. "The extent of missing data for each primary and secondary endpoint will be presented by trial arm along with reasons where available. Details on how missing data will be handled in each analysis is included in the SAP."<br>Can the authors please provide a description of how they intend to handle missing data in the analysis?	**Action: Text amended in manuscript**<br><br>Missing data approaches will be tailored specifically for each primary/secondary analysis - hopefully that will now be conveyed in 'Missing data considerations' section
10. "Primary endpoint analysis will be conducted on the intention-to-treat (ITT) population" [Abstract]<br>and<br>"Primary analysis of the primary outcome measure, time to complete healing will be conducted on the intention-to-treat (ITT) population"<br>Can the authors also consider undertaking a per protocol (PP) sensitivity analysis?	**Action: No change to manuscript**<br><br>Thank you for your comment. Per-protocol analysis requires strong assumptions about the comparability of compliers and non-compliers within randomised arms and is known to be subject to selection bias. Planned supplementary analyses include modelling the causal effect of treatment adherence on the primary outcome measure. This complements the primary analysis in a similar manner to a PP analysis.

11. "Trial status<br>Recruitment started in February 2024 and is expected to complete by December 2025 with a 6-month follow-up period. As of 31st August 2025, 215 participants have been randomised. The full trial protocol is available on the National Institute for Health Research journals library https://fundingawards.nihr.ac.uk/award/NIHR151863"<br>Can start and expected end dates please also be noted by the authors in the Abstract and Methods?	**Action: text partially amended in manuscript**<br><br>Manuscript has been amended with expected end date in 'trial registration number' section of abstract and in 'trial status' section.<br><br>In line with other BMJ Open published protocols and the template, the authors have omitted adding start date as it was confusing where to add in the abstract and given that the registered date is already included in the text. However, the registry has now been updated with both start and end dates in dedicated section of the website.

VERSION 2 - REVIEW

Reviewer 1

Name Epstein, David

Affiliation Universidad de Granada - Campus de Cartuja, Department of Applied Economics

Date 31-Jul-2026

COI

Resource use data collection is stated as "26 weeks (where applicable, if

wound is not healed) " Why not collect resource use at 26 weeks in all participants?

Could you include an quantitative estimate of the proportion who you expect to heal in each arm at 26 weeks, which gives an idea of how important extrapolation will be for the follow-up model.

Reviewer 2

Name Kelson, Zoe

Affiliation University of Exeter, Mathematics

Date 05-Aug-2026

COI

Many thanks to the authors for their responses and revisions.

Author response:

"Study sites are collecting screening data on patients who are eligible but not recruited. The demographics will be compared with the recruited sample of patients. "

Reviewer request:

Can the authors please clarify this response to reviewer within the protocol?

VERSION 2 - AUTHOR RESPONSE

Reviewer Comment	Authors Response
Resource use data collection is stated as "26 weeks (where applicable, if<br>wound is not healed) " Why not collect resource use at 26 weeks in all participants?	Apologies, Resource use data is collected on all participants at week 26.<br>In the section 'Assessments and data collection' it states that Resource use data is collected on all participants at 26 weeks – this is correct.<br>In the section on Questionnaires – the information was incorrect with regard to Resource use data collection – this has been amended.
Could you include an quantitative estimate of the proportion who you expect to heal in each arm at 26 weeks, which gives an idea of how important extrapolation will be for the follow-up model.	Based on sample size assumptions:<br><br>unhealed **healed**<br>SC + CT proportion at 26 weeks 10.8% **89.2%**<br>SC proportion at 26 weeks 21.1% **78.9%**<br><br>This detail has been provided for the reviewer but it was not felt necessary to include in the manuscript.
"Study sites are collecting screening data on patients who are eligible but not recruited. The demographics will be compared with the recruited sample of patients. "<br>Reviewer request:<br>Can the authors please clarify this response to reviewer within the protocol?	The following sentence can be found in the section on Eligibility:<br>Screening logs will be maintained to review reasons not eligible or unable/declined consent and to demonstrate the representativeness of the study sample.
